# Carcinoma Ex Pleomorphic Adenoma of the Palate Composed of Invasive Micropapillary Salivary Duct Carcinoma and Adenoid Cystic Carcinoma Components: An Unusual Case With Immunohistochemical Approach

**DOI:** 10.1097/MD.0000000000000146

**Published:** 2014-12-12

**Authors:** Bruno T. Sedassari, Nelise A. da Silva Lascane, Priscila L. Tobouti, Fernanda M. Pigatti, Maria I.F. Franco, Suzana C.O.M. de Sousa

**Affiliations:** From the Oral Pathology Department (BTS, NADSL, PLT, FMP, COMDS); Pathology Department, School of dentistry/University of São Paulo (MIFF), São Paulo, Brazil.

## Abstract

Carcinoma ex pleomorphic adenoma (CXPA) is an unusual epithelial malignancy that develops from a primary or recurrent pleomorphic adenoma (PA), the most common tumor of salivary glands, and constitutes about 11.5% of all carcinomas that affect these glands. Intraoral minor salivary glands and seromucous glands of the oropharynx are uncommon locations of CXPA. On histopathological examination, the tumor comprises a wide morphological spectrum with a variable proportion between the benign and malignant components with the latter often predominating and overlapping the PA, which may cause misdiagnosis. Here, we report a case of palatal minor salivary gland CXPA composed of invasive micropapillary salivary duct carcinoma and adenoid cystic carcinoma components with multiple nodal metastases in a 74-year-old woman. Neoplastic cells showed heterogeneous immunohistochemical profile with both luminal and myoepithelial differentiation. The invasive micropapillary salivary duct carcinoma component demonstrated overexpression of the oncoprotein human epidermal growth factor receptor-2. This feature should be considered and evaluated as a possible target for adjuvant therapy in case of metastatic disease.

## INTRODUCTION

Carcinoma ex pleomorphic adenoma (CXPA) is defined as an epithelial malignancy that arises in or from a pleomorphic adenoma (PA), the most common tumor of salivary glands.^1,2^ It is an uncommon neoplasm and comprises about 3.6% of all salivary tumors and 11.6% of all salivary gland carcinomas.^3^ The pathogenesis of CXPA remains unclear, but the accumulation of genetic alterations on long-standing tumors seems to be a crucial event in carcinomatous transformation of PA.^4–7^

Under microscopic examination, the tumor comprises a wide morphological spectrum with variable proportion of the malignant component, which varies from a small focus to almost the entire lesion and is often represented by a high-grade adenocarcinoma, not otherwise specified, while specific types of salivary gland carcinomas such as myoepithelial carcinoma, adenoid cystic carcinoma, or salivary duct carcinoma can occur.^1–3,6,8,9^

The involvement of minor salivary glands by CXPA is infrequent, representing about 18% of the cases, mainly the soft and hard palate as the most common topographies.^3^ We describe the morphological and immunohistochemical aspects in a case of invasive micropapillary salivary duct carcinoma (IMSDC) and adenoid cystic carcinoma (ACC) as malignancies arising in a PA of the palate in a 74-year-old female patient with multiple nodal metastasis.

## CASE REPORT

A 74-year-old white woman presented with a painful swelling in the right hard and soft palates associated with enlarged homolateral regional lymph nodes. A malignant salivary gland tumor was suspected and the patient underwent resection of the mass with right radical neck dissection. Tumor-free margins were confirmed by microscopic examination of intraoperative frozen sections.

## PATHOLOGY

**FIGURE 1:**
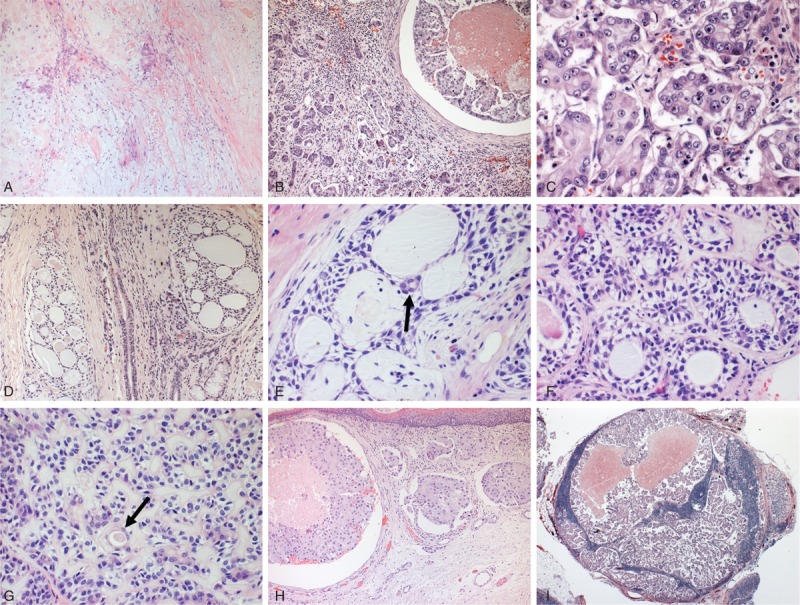
(A) Histological appearance of the remaining pleomorphic adenoma withtubular structures in a myxochondroid background (hematoxylin and eosin, ×40). (B) Intraductal (right) and invasive (left) areas of salivary duct carcinoma component (×40). (C) Invasive area of the salivary duct carcinoma consisted of cell clusters without fibrovascular cores surrounded by clear spaces separating them from the stroma (×100). (D) The adenoid cystic component consisted predominantly of cribriform structures (×40) with pseudocystic spaces lined by the neoplastic cells (E) and occasional eosinophilic cuboidal cells lining luminal spaces (arrow) (×100). (F) Double-layered tubules in adenoid cystic areas and foci of intense basal lamina deposition (G) associated with eosinophilic cuboidal cells determinig ductiform spaces (arrow) (×100). (H) Massive carcinomatous embolization of mucosal blood and lymphatic vessels (×40). (I) Metastatic foci in lymph node exclusively composed by the invasive micropapillary salivary duct carcinoma with areas of coagulative necrosis (×25).

The resected specimen and 79 cervical lymph nodes were fixed in 4% buffered formalin and paraffin-embedded. Microscopic examination revealed an invasive neoplastic process with 3 distinct and well-demarcated recognized elements. The first element, the scarcest area, was typical of PA, represented by occasional bilayered duct-like structures in association with a myxochondroid stroma, which showed areas of discrete hyalinization (Figure 1A). The second included intraductal and invasive areas of salivary duct carcinoma (Figure 1B), the latter with a micropapillary architecture. The intraductal areas were composed of cells with abundant eosinophilic cytoplasm, pleomorphic nuclei with conspicuous nucleoli arranged in nests of varying size in a Roman bridge-like cribriform pattern with central comedonecrosis. Morula-like cell clusters without fibrovascular cores represented the invasive micropapillary component, which was surrounded by clear spaces that separated them from the inflammatory stroma (Figure 1C). The third element had appearance of ACC with predominantly cribriform and double-layered tubular structures lined by small cells with scant cytoplasm, hypercromatic and angulated nuclei (Figure 1D–F). There were extensive foci of intense basal lamina production in some areas and eosinophilic cuboidal cells lining tubular structures were present (Figure 1G). Multiple tumor emboli could be observed in mucosal lymphatic and blood vessels (Figure 1H). Thirty-nine lymph nodes showed metastatic deposits composed exclusively of IMSDC component (Figure 1I). Fourteen months after tumor resection, the patient is alive and without evidence of disease.

**FIGURE 2:**
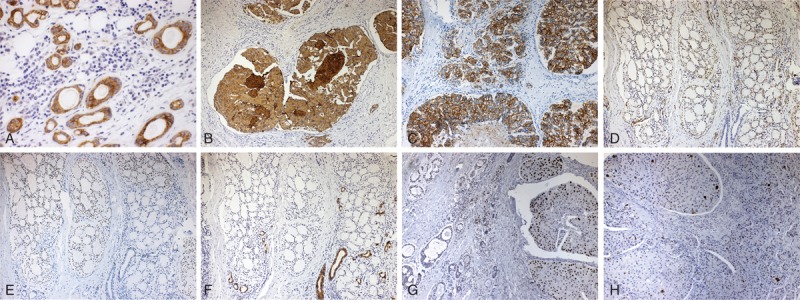
(A) Immunohistochemically, ductal cells of the remaining pleomorphic adenoma expressed CK7 (×100). (B) The invasive micropapillary salivary duct carcinoma cells strongly stained with CK7 and HER-2 in a membrane pattern (C), both in intratubular and invasive areas (×100). (D) Modified myoepithelial cells of the adenoid cystic carcinoma component expressed α-SMA, nuclear p63 (E), and CK7 (F) highlighted cells with luminal differentiation (×40). (G) p53 expression in IMSDC (right) and in ACC (left) (×100). (H) Ki-67 expression in malignant neoplastic cells (×100). ACC = adenoid cystic carcinoma, HER-2 = human epidermal growth factor receptor-2, IMSDC = invasive micropapillary salivary duct carcinoma, α-SMA = α-smooth mucle actin.

Immunohistochemical stains showed that in residual PA area, cytokeratin (CK)7 protein was positive in pseudoluminal-forming cells (Figure 2A); the IMSDC component cells had a strong cytoplasmic positivity to CK7 and to human epidermal growth factor receptor 2 (HER-2) in a membrane pattern, both in intraductal and invasive micropapillary areas (Figure 2B and C). Cells with myoepithelial differentiation in ACC were positive to α-smooth mucle actin (α-SMA) and p63, as well as CK7 highlighted those cells that determined luminal spaces (Figure 2D–F). The c-kit protein was negative. Both carcinomatous components displayed diffuse nuclear p53 immunoexpression (Figure 2G), whereas the PA was negative. Ki-67 was expressed in <50% of the neoplastic cells in IMSDC, whereas the ACC showed a very low proliferative index, with about 5% of Ki-67 expression (Figure 2H).

## DISCUSSION

CXPA is an infrequent malignant tumor of the salivary glands historically named as malignant mixed tumor, carcinoma ex mixed tumor, carcinoma ex adenoma, and carcinoma ex benign pleomorphic adenoma.^1^ Although most CXPA occur in major salivary glands, cases involving the oral and oropharyngeal minor glands account for about 17.5% of the Armed Forces Institute of Pathology series. Of all these cases, 63% were in the palate and 10.5% were in the upper lip. Other sites included the tongue, buccal mucosa, tonsil, and oropharynx. Signs and symptoms most commonly reported were a painless mass of long duration.^10^

Although carcinomas arising in PA represent a wide morphological spectrum, in most cases, CXPA is usually a high-grade adenocarcinoma, not otherwise specified, but other differentiated salivary gland malignancies such as mucoepidermoid carcinoma, squamous cell carcinoma, ACC, adenosquamous carcinoma, epithelial-myoepithelial carcinoma, acinic cell carcinoma, clear cell carcinoma, myoepithelial carcinoma, and sarcomatoid carcinoma have been described.^3,11,12^ The double differentiation of the carcinomatous component of CXPA described in this case is a very exceptional event and has been previously reported by 2 groups: Freeman et al^13^ described a fatal nasal CXPA with adenoid cystic and squamous carcinomatous differentiation and Nakamori et al^14^ an intracapsular salivary duct and squamous cell CXPA of the buccal mucosa (Table 1). So far, no case of palatal CXPA composed of IMSDC and ACC has been reported.

**TABLE 1:**
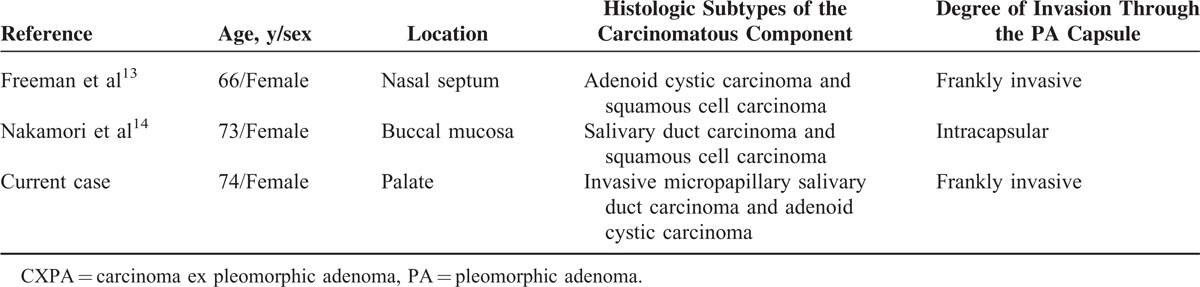
Reported Cases of CXPA With Double Differentiation of the Carcinomatous Component

Based on the degree of extension through the capsule of “maternal” PA by the carcinoma, CXPA may be subclassified as intracapsular, noninvasive or in situ, minimally invasive (<1.5 mm penetration of the malignant component into extracapsular tissue), and frankly invasive (>1.5 mm of invasion from the tumor capsule into the adjacent tissue) with prognostic implications.^1,11,12,15^ Frankly invasive CXPA is often associated with metastatic disease and poor prognosis.^15,16^ In contrast, intracapsular and minimally invasive carcinomas have been considered tumors that behave in a similar fashion to PA without malignant transformation.^16,17^ This statement is confirmed in this case, in which both carcinomatous components were of the frankly invasive subtype and associated with the presence of multiple nodal metastasis.

As would be expected, modified myoepithelial cells of the ACC component showed diffuse immunoreactivity to the myoepithelial markers p63 and α-SMA, whereas CK7 was positive in those cells with a luminal distribution and c-kit was negative. The c-kit protein has been considered as a helpful marker in distinction between ACC and other salivary gland tumors, which share morphological similarities, such as polymorphous low-grade adenocarcinoma.^18,19^ The negative stain found should be partially explained by the fact that the ACC arising in a previous PA probably displays a different immunoprofile and genetic abnormalities than ACC without PA association, perhaps owing to different mechanisms of carcinomatous initiation and progression, despite the similar histopathological aspects. However, the p53 oncoprotein showed diffuse positive staining in carcinomatous areas, but not in residual PA. The p53 oncoprotein has been implicated in the pathogenesis of CXPA, and our findings corroborate this hypothesis. Furthermore, p53 overexpression is considered a helpful tool in identifying areas of carcinomatous transformation in PA.^20^

The HER-2 oncoprotein is extensively studied in mammary ductal invasive carcinoma and its overexpression is often associated with a poor prognosis and high frequency of nodal metastasis.^21^ Nevertheless, it is considered to play an important role in targeting adjuvant therapy,^22^ as well as has been employed as an immunohistochemical marker to identify areas of malignant transformation in PA, particularly if the carcinoma is of the salivary duct type.^23^ Furthermore, HER-2 overexpression by immunohistochemistry or amplification by fluorescent in situ hybridization test in salivary gland CXPA and other salivary gland malignancies may elect patients for Trastuzumab therapy, especially in case of metastatic disease.^23^ Recently, Di Palma et al^24^ reported a case of plurimetastatic CXPA successfully treated by Trastuzumab and radiotherapy. In the presented case, HER-2 overexpression was identified only in the IMSDC component by immunohistochemistry and curiously the 39 nodal metastatic foci were composed exclusively by this tumor. If the patient experience recurrence, visceral metastasis, or both by the IMSDC, the status of HER-2 observed in this case could call attention to the possibility of adjuvant therapy with Trastuzumab to cure.

In summary, we report an unusual case of palatal minor salivary gland CXPA composed of IMSDC and ACC with extensive nodal involvement based on histopathological and immunohistochemical findings. Improved understanding of the pathobiology of this tumor and other salivary gland tumors may lead to rationally designed targeted therapies that could improve the outcome of patients with CXPA.
